# Case Report: Focal nodular hyperplasia of the liver in an adolescent—a case report and literature review

**DOI:** 10.3389/fped.2026.1673584

**Published:** 2026-06-16

**Authors:** Rongchun Xing, Yuhong Wen, Dan Yu, Chongyuan Chen, Mingzheng Hu

**Affiliations:** 1 The First College of Clinical Medical Science, China Three Gorges University, Yichang, Hubei, China; 2 Department of Hepatobiliary Surgery, Yichang Central People’s Hospital, Yichang, Hubei, China

**Keywords:** child, FNH, focal nodular hyperplasia, HCA, hepatocellular adenoma, outcomes

## Abstract

Focal nodular hyperplasia (FNH) of the liver is not a true neoplasm but rather a hyperplastic response to localized vascular malformations. In women of reproductive age, it is typically discovered incidentally and managed conservatively due to its benign nature. However, in adolescents, FNH demonstrates marked clinical heterogeneity. We present the case of a 17-year-old female patient with a hepatic lesion that increased in size to 6.0 cm within four months, exhibiting ambiguous imaging features. The central diagnostic dilemma lies in the need to differentiate FNH from hepatocellular adenoma (HCA), particularly high-risk subtypes, which may mimic classic imaging features of FNH while carrying significant risks of hemorrhage and malignant transformation. In this case, due to the inability to definitively exclude HCA through non-invasive modalities, laparoscopic partial hepatectomy was performed, and postoperative pathology confirmed FNH. A comprehensive literature review underscores that FNH exhibits substantial heterogeneity across the human lifespan, challenging the applicability of adult-centric management paradigms. Therefore, for adolescents with atypical hepatic lesions exceeding 5 cm and indeterminate imaging findings, proactive surgical intervention may offer definitive diagnosis and curative treatment, safeguarding long-term patient outcomes.

## 1 Introduction

Focal nodular hyperplasia (FNH) is the second most common benign hepatic lesion after cavernous hemangioma, with an estimated prevalence of 0.9% to 3.0% in the general population (1). It is not a true neoplasm but rather a polyclonal hyperplastic response of hepatocytes to focal vascular anomalies. The classic clinical and radiological presentation of FNH is predominantly seen in women of reproductive age, typically between 20 and 50 years old, with a female-to-male ratio as high as 8:1 (2). In adults, FNH is generally asymptomatic and most often detected incidentally during imaging performed for unrelated reasons (3). Given its extremely low risk of malignant transformation and rare complications such as spontaneous rupture or hemorrhage, international consensus guidelines recommend conservative observation in asymptomatic adults with confirmed diagnoses (4).

However, FNH presents significant variations in clinical behavior, diagnostic strategies, and therapeutic approaches across different age groups (5). In children and adolescents, FNH is rare, accounting for only 2%–7% of all primary hepatic tumors in the pediatric population (6). Unlike the predominantly silent presentation in adults, more than half of pediatric patients experience symptoms such as abdominal pain or a palpable mass at diagnosis (7). Moreover, pediatric FNH often lacks typical radiologic features, such as the central stellate scar, making non-invasive diagnosis more challenging (8). These age-related differences are central to understanding the complexity of FNH (9).

Diagnostic uncertainty is particularly pronounced in adolescent patients (10). The primary challenge lies in accurately distinguishing FNH from hepatocellular adenoma (HCA), a monoclonal neoplasm associated with significant clinical risks (11). HCA carries a 10%–27% risk of spontaneous hemorrhage and a 5%–10% risk of malignant transformation to hepatocellular carcinoma (HCC), risks that escalate significantly when the lesion exceeds 5 cm in diameter (12). The present case—a 17-year-old female with a hepatic lesion that rapidly grew to 6.0 cm and exhibited ambiguous imaging findings—highlights this high-risk diagnostic dilemma (13).

This report presents a detailed account of a diagnostically challenging adolescent FNH case, contextualized through a literature review spanning pediatric, adult, and geriatric populations. By comparing clinical presentations, imaging characteristics, and management strategies across age groups and contrasting them with features of HCA, we demonstrate why the conservative management approach derived from adult experience is often inappropriate for adolescents with atypical hepatic lesions. When imaging fails to rule out high-risk tumors, surgical intervention becomes a prudent and necessary clinical decision to ensure optimal patient outcomes.

## 2 Case report

The patient was a 17-year-old female who was admitted to our hospital in June 2025 with a 4-month history of a hepatic space-occupying lesion discovered incidentally during routine health screening. Abdominal ultrasound performed at a local hospital revealed a hepatic mass, prompting referral for further evaluation. The patient was admitted to our facility under the working diagnosis of a hepatic lesion.

She had no history of hypertension, diabetes, viral hepatitis, tuberculosis, or other infectious diseases. She denied tobacco and alcohol use. Past medical history included unspecified cervical surgery. Her general health status was otherwise unremarkable.

On physical examination, vital signs were stable: temperature 36.6 °C, heart rate 59 bpm, blood pressure 110/62 mmHg. She was alert, without scleral icterus. The abdomen was soft and flat, with no tenderness, rebound tenderness, or palpable hepatosplenomegaly. No costovertebral angle tenderness or shifting dullness was noted.

Laboratory investigations revealed normal tumor markers: alpha-fetoprotein (AFP) 3.7 ng/mL, carcinoembryonic antigen (CEA) 1.7 ng/mL, and carbohydrate antigen 19–9 (CA19–9) 31.0 U/mL. Review of imaging studies included:An ultrasound performed on February 7, 2025, which showed a slightly hyperechoic lesion in the liver (5.0 × 4.5 cm), initially considered suggestive of hepatic hemangioma.On June 13, 2025, contrast-enhanced MRI of the upper abdomen revealed a hypervascular lesion in the right hepatic lobe (6.0 × 3.7 cm) with associated arteriovenous shunting, raising suspicion for hepatocellular adenoma (Figure 1).A subsequent contrast-enhanced CT scan also demonstrated a hypervascular lesion in the right hepatic lobe, favoring a diagnosis of FNH, though hepatocellular adenoma could not be definitively excluded (Figure 2).Given the patient’s young age, progressive lesion enlargement during follow-up, and indeterminate imaging findings with a non-negligible possibility of HCA, a multidisciplinary discussion was held. After comprehensive preoperative evaluation and informed consent regarding surgical indications and associated risks (e.g., intraoperative bleeding, bile leakage, adjacent organ injury), laparoscopic partial hepatectomy was planned and performed.

**Figure 1:**
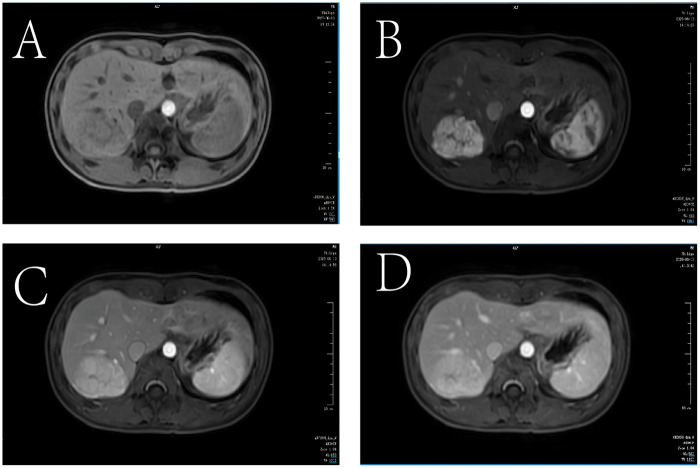
Contrast-enhanced magnetic resonance imaging (MRI) of the upper abdomen on June 13, 2025. (**A**) Axial T1-weighted pre-contrast image showing a hypointense lesion in the right hepatic lobe. (**B**) Axial T2-weighted image demonstrating heterogeneous signal intensity of the lesion. (**C**) Arterial phase post-contrast image showing marked hypervascular enhancement of the lesion (6.0 × 3.7 cm) with associated arteriovenous shunting. (**D**) Portal-venous / delayed phase image showing the lesion. The imaging features raised the suspicion of hepatocellular adenoma (HCA), although focal nodular hyperplasia (FNH) could not be excluded.

**Figure 2:**
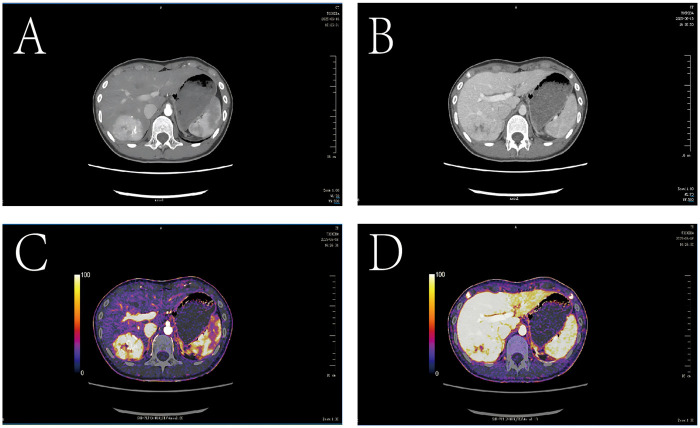
Contrast-enhanced computed tomography (CT) of the upper abdomen. (**A,B**) Axial pre-contrast and arterial phase CT images demonstrating a hypervascular lesion in the right hepatic lobe (segment VII). (**C,D**) Corresponding color-coded CT perfusion maps with the gradient scale on the left indicating perfusion values from 0 to 100, showing increased perfusion within the lesion. The findings favoured a diagnosis of FNH, although HCA could not be definitively excluded.

Notably, preoperative percutaneous biopsy was not performed in this case. This decision was based on several considerations. First, the diagnostic yield of percutaneous biopsy for differentiating FNH from HCA is limited by sampling error and histological overlap, particularly in lesions with atypical features; prior series have reported non-diagnostic or inconclusive biopsy results in up to 30%–40% of hepatocellular lesions. Second, the lesion’s location in segment VII of the right posterior hepatic lobe posed technical challenges for safe percutaneous access, with an attendant risk of hemorrhage from a hypervascular mass. Third, as proposed in the management flowchart by Zarfati et al. in a series of 50 pediatric FNH cases (14), when imaging remains inconclusive and the lesion exceeds 5 cm with documented growth, direct surgical resection is recommended over biopsy, as it simultaneously provides definitive histopathological diagnosis and therapeutic excision. Given that the multidisciplinary team had already determined that surgical intervention was indicated on clinical grounds, percutaneous biopsy was deemed unlikely to alter the management plan and would have introduced additional procedural risk without commensurate diagnostic benefit.

In summary, the diagnostic assessment followed a systematic approach: initial ultrasonography identified the lesion, serial imaging documented progressive growth (from 5.0 cm to 6.0 cm over four months), contrast-enhanced MRI and CT provided conflicting differential diagnoses (HCA vs. FNH), and serum tumor markers (AFP, CEA, CA19–9) were within normal limits. The key diagnostic challenge lay in the overlapping imaging features of FNH and HCA in this age group, where typical FNH hallmarks (central scar, spoke-wheel vascularity) are frequently absent. Given the lesion size exceeding the 5 cm threshold for high-risk HCA, the documented growth trajectory, and the inability to exclude a neoplasm with hemorrhagic and malignant potential through non-invasive means, the multidisciplinary team determined that surgical intervention was both diagnostically and therapeutically indicated The complete chronology of clinical events, from the initial detection of the lesion to postoperative follow-up, is summarized in Table 1.

**Table 1:** Timeline of clinical events.

Date	Event	Key Findings
February 7, 2025	Routine health screening	Abdominal ultrasound: slightly hyperechoic hepatic lesion (5.0×4.5 cm); initially suspected hemangioma
February–June 2025	Observation period (4 months)	Progressive lesion enlargement documented on serial imaging
June 13, 2025	Contrast-enhanced MRI	Hypervascular lesion in right hepatic lobe (6.0×3.7 cm) with arteriovenous shunting; HCA suspected
June 2025	Contrast-enhanced CT	Hypervascular lesion favoring FNH; HCA could not be definitively excluded
June 2025	Laboratory workup	AFP 3.7 ng/ml, CEA 1.7 ng/ml, CA19-9 31.0 U/ml (all within normal limits)
June 2025	Multidisciplinary discussion	Decision for surgical intervention due to diagnostic uncertainty, progressive growth, and inability to exclude HCA
June 2025	Laparoscopic partial hepatectomy	Complete resection of segment VII lesion (6.0×4.0 cm); uneventful procedure
Postoperative	Histopathological examination	Diagnosis of FNH confirmed by characteristic microscopic findings and immunohistochemistry
1 week postoperative	Hospital discharge	Uneventful recovery; no residual discomfort
Follow-up	Outpatient review	No abnormalities detected; patient reported satisfactory quality of life with full resumption of daily activities

Under general anesthesia, the patient was placed in a supine position, and routine sterile preparation was completed. A laparoscopic approach was used to establish operative access. Intraoperative exploration revealed no ascites. The liver surface appeared smooth and soft, with no signs of cirrhosis. The lesion was located in segment VII (S7) of the right posterior hepatic lobe, measuring approximately 6.0 × 4.0 cm.

Intraoperative gross photography of the resected specimen was not obtained, as the specimen required immediate submission for intraoperative frozen-section pathological examination under strict aseptic conditions; thus, imaging-based characterization (Figures 1, 2) constitutes the primary visual documentation of the lesion.

The lesion was completely excised with adequate margins, and meticulous hemostasis was confirmed. The operation was completed uneventfully, and the patient was transferred to the recovery unit.

Postoperatively, the patient recovered uneventfully without major complications and was discharged one week after surgery in stable condition. The postoperative course was characterized by early mobilization on postoperative day 1, resumption of oral intake on postoperative day 2, and progressive return to normal diet by postoperative day 4. Postoperative analgesia was managed with intravenous paracetamol and oral non-steroidal anti-inflammatory drugs; no opioid analgesia was required. Drain output was minimal (serous fluid, less than 50 mL/day) and the abdominal drain was removed on postoperative day 5. Serial liver function tests demonstrated a transient mild elevation of transaminases (peak alanine aminotransferase 85 U/L on postoperative day 1) with normalization by postoperative day 4. No bile leakage, postoperative hemorrhage, wound infection, or thromboembolic events were observed.

At follow-up visits, the patient reported no residual discomfort and expressed satisfaction with the surgical outcome. She had resumed all normal daily activities, including school attendance and physical exercise, without any limitations. The patient and her family reported relief at the definitive histopathological diagnosis, which eliminated the uncertainty and anxiety associated with a potentially malignant hepatic lesion. Overall, the patient’s self-assessed quality of life was satisfactory and comparable to her pre-illness baseline. Specifically, at the two-week postoperative visit, the surgical wounds were well-healed with no evidence of infection or incisional hernia. Liver function tests (alanine aminotransferase, aspartate aminotransferase, total bilirubin, and albumin) had returned to within normal reference ranges. At the three-month follow-up, contrast-enhanced abdominal ultrasound demonstrated a normal postoperative hepatic parenchyma with no evidence of residual or recurrent lesion at the resection site. The patient tolerated a regular diet without gastrointestinal complaints, and her body weight remained stable. No adverse or unanticipated events were observed during the entire follow-up period. A structured long-term surveillance protocol has been established, consisting of abdominal ultrasound and liver function tests every six months for the first two years, followed by annual imaging thereafter, in accordance with current recommendations for post-resection monitoring of benign hepatic lesions.

Histopathological examination of the resected specimen confirmed the diagnosis of focal nodular hyperplasia (FNH), based on characteristic microscopic findings and immunohistochemical staining patterns.

## 3 Discussion

Focal nodular hyperplasia (FNH) is widely regarded pathologically as a non-neoplastic, polyclonal hyperplastic response of hepatocytes to localized vascular abnormalities. In typical adult cases, asymptomatic lesions with classic imaging characteristics are often managed conservatively due to their negligible risk of malignant transformation or complications. However, the case described in this report—a 17-year-old female with a hepatic lesion that showed progressive growth and was ultimately confirmed as FNH through surgical resection—represents a significant deviation from conventional diagnostic and management paradigms. This case exemplifies the central premise raised in the introduction: FNH is not a homogeneous disease entity, and its clinical presentation, etiology, and management strategies vary significantly throughout the life course.

In adults, particularly women of reproductive age, FNH presents with the most classic and well-characterized features. The condition exhibits a marked female predominance (female-to-male ratio approximately 8:1). Most adult FNH lesions are discovered incidentally during imaging performed for unrelated reasons, with patients remaining entirely asymptomatic (2, 15). Although a possible association with oral contraceptives (OCPs) has been historically suggested, current consensus holds that exogenous estrogens may accelerate the growth of preexisting lesions rather than initiate their formation (16). Imaging findings in adults are typically characteristic—such as a central scar, homogeneous arterial-phase enhancement, and absence of a capsule—which confer high diagnostic accuracy via non-invasive modalities and justify conservative management as the standard of care (17).

In contrast, the clinical profile of our adolescent patient deviates considerably from that of the adult paradigm (18). Her age was well below the typical range of FNH onset, and the lesion exhibited progressive enlargement, reaching 6.0 cm—features that are uncommon in incidentally detected adult FNH (4).

Among pediatric and adolescent populations, FNH is a rare hepatic tumor, accounting for only 2%–7% of all liver neoplasms in children (19). Both clinical and radiologic features differ markedly from those in adults (20). Pediatric FNH is more likely to be symptomatic, with approximately one-third of children presenting with abdominal pain (21). Lesions tend to be larger at diagnosis (one study found that 64% exceeded 5 cm in diameter), and the incidence of multifocal disease is higher than in adults (22). Moreover, atypical imaging features are the norm rather than the exception in pediatric cases—central scars are seen less frequently, and two-thirds of lesions may lack typical radiological hallmarks (23). Etiologically, pediatric FNH has a stronger association with specific risk factors, such as prior exposure to hepatotoxic chemotherapy and/or radiotherapy for childhood malignancies, as well as congenital vascular anomalies like portosystemic shunts or biliary atresia. Although our patient presented with a large lesion consistent with pediatric FNH, she lacked any of the known risk factors, rendering the case atypical even within this subgroup and further complicating diagnosis (24).

While rare, FNH has also been reported in elderly individuals, reinforcing its presence across the entire human lifespan (25). For instance, a case involving a 79-year-old male demonstrated lesion onset in late life, with subsequent progression in both size and number over a decade (26). This challenges the long-held notion of FNH as a condition limited to women of childbearing age, and underscores the dynamic and heterogeneous nature of FNH over time (27). Such variability necessitates that clinicians move beyond age-specific diagnostic assumptions when evaluating atypical presentations (28).

The most critical diagnostic challenge in this adolescent case was the need to differentiate FNH from hepatocellular adenoma (HCA), given the stark contrast in prognosis and management strategies between the two entities (Table 2) (29).

**Table 2:** Differentiating Features of FNH and HCA Subtypes.

Feature	Focal Nodular Hyperplasia (FNH)	H-HCA (HNF1A-inactivated HCA)	I-HCA (Inflammatory HCA)	b-HCA (β-catenin activated HCA)
Pathophysiology	Polyclonal hyperplastic response	Monoclonal tumor	Monoclonal tumor	Monoclonal tumor
Key Risk Factors	Vascular malformation, OCPs (growth accelerator)	OCPs, MODY3 (maturity-onset diabetes of the young type 3)	Obesity, alcohol, metabolic syndrome	Male sex, androgens, glycogen storage disease
Malignant Potential	None	Very low (if <5 cm)	Low (unless β-catenin mutated)	High (∼40%)
Hemorrhagic Risk	Rare	Moderate	High (∼30%)	Moderate
MRI T1/T2 Signals	T1: iso-/hypointense; T2: iso-/hyperintense	T1: variable, often hyperintense	T2: markedly hyperintense ("atoll sign")	Heterogeneous, nonspecific
Central Scar	∼50%, T2 hyperintense	Absent	Absent	Absent
Intralesional Fat	Rare	Characteristic feature (signal drop in opposed-phase imaging)	Possible	Rare
Hepatobiliary Phase	Iso-/hyperintense	Markedly hypointense	Hypointense (but may mimic FNH)	>50% iso-/hyperintense

FNH is a benign hyperplastic lesion with virtually no risk of malignant transformation or complications (30). In contrast, HCA is a monoclonal neoplasm with substantial clinical risks—including spontaneous rupture and hemorrhage (10%–27%) and malignant transformation into hepatocellular carcinoma (HCC) (5%–10%) (10). These risks are especially pronounced in lesions >5 cm, male patients, and specific molecular subtypes (31). The lesion in our patient measured 6.0 cm with documented growth, thus fulfilling criteria for high-risk HCA if it were indeed of neoplastic origin (32). Consequently, the inability to exclude HCA based on imaging alone redefined the clinical context: this was no longer the management of a benign “incidentaloma,” but rather a scenario necessitating proactive mitigation of oncologic and hemorrhagic risk.

Molecular heterogeneity within HCA further complicates diagnostic efforts (33). The *β*-catenin activated subtype (b-HCA) is of particular clinical concern due to its high malignant potential—transformation rates may reach 40% (34). A major diagnostic pitfall arises from the fact that certain HCA subtypes—particularly the inflammatory subtype (I-HCA) and some high-risk b-HCA variants—may mimic FNH on hepatocyte-specific contrast-enhanced MRI (e.g., with Gd-EOB-DTPA), exhibiting contrast retention during the hepatobiliary phase, a hallmark traditionally associated with FNH. This imaging “mimicry” introduces diagnostic ambiguity that cannot be definitively resolved without histopathological examination of the entire lesion.

In clinical practice, a structured diagnostic algorithm is essential for distinguishing FNH from HCA and other hepatic lesions. The initial evaluation should include serum biomarkers: alpha-fetoprotein (AFP) elevation, although typically associated with hepatocellular carcinoma (HCC), may also occur in certain HCA subtypes and serves as a useful screening tool; in our patient, AFP was within normal limits (3.7 ng/mL), which reduced but did not eliminate the possibility of malignancy. Additional biomarkers such as CEA and CA19–9 further aid in excluding metastatic or cholangiocarcinoma etiologies. Contrast-enhanced MRI with hepatocyte-specific agents (Gd-EOB-DTPA) remains the cornerstone of non-invasive differentiation, as FNH characteristically demonstrates iso- or hyperintensity during the hepatobiliary phase due to functioning hepatocytes and intact biliary structures, whereas most HCA subtypes appear hypointense. However, as demonstrated in our case, this criterion is not absolute—inflammatory HCA (I-HCA) and certain *β*-catenin-activated variants may exhibit paradoxical hepatobiliary phase uptake, mimicking FNH. Contrast-enhanced ultrasound (CEUS) may serve as a complementary modality, with FNH typically showing rapid centrifugal arterial enhancement and a characteristic spoke-wheel pattern. When non-invasive modalities remain inconclusive—particularly in lesions exceeding 5 cm with documented growth—percutaneous biopsy may be considered, although its diagnostic yield for distinguishing FNH from HCA is limited by sampling error and histological overlap. In our case, preoperative biopsy was deliberately omitted for the following reasons: (1) the lesion’s deep location in segment VII presented technical challenges for safe percutaneous access; (2) the hypervascular nature of the mass increased the risk of hemorrhagic complications from a needle biopsy; (3) the well-recognized limitations of core biopsy in distinguishing FNH from HCA—including sampling error, tissue fragmentation, and the inability to assess overall lesion architecture—meant that a non-diagnostic result would not alter the management plan; and (4) given that surgical intervention was already indicated based on the clinical scenario (progressive growth, diagnostic uncertainty, lesion size >5 cm), direct excisional biopsy via resection was the most rational approach. This rationale is consistent with the management flowchart proposed by Zarfati et al. (14), which recommends surgical resection over biopsy for pediatric hepatic lesions that remain indeterminate after comprehensive imaging workup. In such scenarios, surgical resection remains the gold standard for definitive diagnosis, simultaneously providing curative treatment if the lesion proves to be neoplastic.

Allowing a growing, undiagnosed lesion with non-negligible risk of being a high-risk HCA to remain in a young patient would jeopardize long-term health outcomes (35). Thus, laparoscopic resection of the segment VII lesion was performed (36). Laparoscopic liver resection (LLR) was selected to minimize procedural morbidity (37). Compared to open hepatectomy, LLR offers well-documented advantages, including reduced intraoperative blood loss, lower complication rates, less postoperative pain, shorter hospital stay, and faster return to normal activities—all contributing to improved postoperative quality of life (38). The minimally invasive approach allowed the surgical team to achieve complete lesion resection while minimizing physiological stress, facilitating rapid and complete recovery for this young patient (39).

The therapeutic decision-making in this case adhered to established principles of individualized surgical oncology. The choice of laparoscopic partial hepatectomy over open surgery was guided by the lesion's location in segment VII, its well-circumscribed nature on preoperative imaging, and the patient's young age and desire for optimal cosmetic outcomes. No changes in the intervention plan were required during the procedure, as intraoperative findings were consistent with preoperative imaging assessments.

This case report has several strengths and limitations that merit consideration. The principal strengths include the comprehensive multimodal imaging workup (ultrasound, contrast-enhanced MRI, and CT), the minimally invasive surgical approach that balanced diagnostic necessity with procedural safety, and the definitive histopathological confirmation of the diagnosis. Furthermore, the systematic comparison of FNH behavior across pediatric, adult, and geriatric populations provides a clinically relevant framework for individualized management. However, several limitations should be acknowledged. First, as a single-case report, the generalizability of our findings is inherently limited. Second, hepatocyte-specific contrast agents (Gd-EOB-DTPA) were not utilized in this patient, which might have enhanced the diagnostic accuracy of MRI. Third, the follow-up period is relatively short, and long-term outcomes data are not yet available. Finally, molecular or genetic characterization of the resected lesion was not performed, which could have provided additional insights into the pathogenesis of FNH in this atypical demographic.

The diagnostic journey in this case serves as a compelling illustration of how atypical features in adolescent FNH necessitate departure from standard adult-oriented management strategies (40). The lesion’s continuous enlargement and ambiguous imaging characteristics created a clinical impasse in which the foremost priority became exclusion of high-risk HCA (41). This justified the decision to proceed with surgery, despite eventual confirmation of a benign lesion (14). Surgical resection not only provided diagnostic certainty but also eliminated any potential oncologic threat—underscoring its value as the optimal strategy when imaging alone cannot resolve diagnostic uncertainty in hepatic masses (42, 43).

## 4 Conclusion

This case demonstrates that FNH exhibits significant clinical heterogeneity across the lifespan, and that adult-derived conservative management paradigms cannot be universally applied. When imaging cannot reliably exclude hepatocellular adenoma—particularly in adolescents with growing lesions exceeding 5 cm—proactive, minimally invasive surgical intervention offers both definitive histopathological diagnosis and long-term cure, representing the most prudent clinical strategy.

## Data Availability

The original contributions presented in the study are included in the article/supplementary material, further inquiries can be directed to the corresponding authors.
